# Association between maternal and fetal weight gain: cohort study

**DOI:** 10.1590/S1516-31802012000400007

**Published:** 2012-09-04

**Authors:** Bárbara Miranda Ferreira Costa, Régis Resende Paulinelli, Maria Alves Barbosa

**Affiliations:** I MSc. Nutritionist, Department of Nutrition, Universidade Paulista (Unip), Goiânia, Brazil.; II MD, PhD. Gynecologist, Department of Gynecology and Obstetrics, Medical School, Universidade Federal de Goiás (UFG), Goiânia, Brazil.; III PhD. Nurse, Nursing School, Universidade Federal de Goiás (UFG), Goiânia, Brazil.

**Keywords:** Body mass index, Feeding behavior, Fetal macrosomia, Weight gain, Pregnancy, Índice de massa corporal, Comportamento alimentar, Macrossomia fetal, Ganho de peso, Gravidez

## Abstract

**CONTEXT AND OBJECTIVE:**

Excessive gestational weight gain is related to many complications (both maternal and fetal), such as macrosomia. The most common complications in macrosomic fetuses include: increased risk of intrauterine death, need for intensive care, fractures, neonatal hyperbilirubinemia, paralysis of the brachial plexus and obesity in childhood and adulthood. The aim of this study was to evaluate the association between gestational and fetal weight gain and the incidence of macrosomia in two maternity hospitals.

**DESIGN AND SETTING:**

Cohort study in two public maternity hospitals in Goiânia, Brazil.

**METHODS:**

This was a cohort study on 200 healthy pregnant women with normal body mass index, divided into two groups: one with normal weight gain and the other with excessive weight gain during pregnancy.

**RESULTS:**

The cohorts were similar regarding maternal age, per capita income, schooling level and reproductive behavior. The fetal weight was greater in the cohort with excessive maternal weight gain (3,388.83 g ± 514.44 g) than in the cohort with normal weight (3,175.86 g ± 413.70 g) (P < 0.01). The general incidence of macrosomia was 6.5%: 13.0% (13 cases) in the cohort with excessive maternal weight gain and 0.0% (0 cases) in the cohort with adequate weight gain.

**CONCLUSION:**

Excessive maternal weight gain was associated with increased fetal birth weight and incidence of macrosomia.

## RESUMO

**CONTEXTO E OBJETIVO:**

O ganho de peso gestacional excessivo está relacionado a inúmeras complicações tanto maternas como fetais, como por exemplo, a macrossomia. Esta, por sua vez, pode aumentar o risco de morte intra-uterina, necessidade de cuidados intensivos, fraturas, hiperbilirrubinemia neonatal, paralisia do plexo braquial e obesidade na infância e fase adulta. O objetivo deste estudo foi avaliar a associação do ganho de peso gestacional com o ganho de peso fetal e a incidência de macrossomia em duas maternidades.

**TIPO DE ESTUDO E LOCAL:**

Estudo de coorte em duas maternidades públicas em Goiânia, Brasil.

**MÉTODOS:**

Estudo de coorte com 200 gestantes saudáveis com índice de massa corporal normal, divididas em dois grupos, um com ganho de peso adequado e o outro com peso excessivo na gravidez.

**RESULTADOS:**

As coortes foram semelhantes quanto à idade materna, renda per capita, escolaridade e comportamento reprodutivo. O peso fetal foi maior na coorte de ganho de peso materno excessivo (3388,83 g ± 514,44 g) do que na de peso normal (3175,86 g ± 413,70 g) (P < 0,01). A incidência geral de macrossomia foi 6,5% sendo de 13,0% (13 casos) na coorte com ganho de peso materno excessivo e de 0,0% (0 casos) na de peso adequado.

**CONCLUSÃO:**

O ganho de peso materno excessivo esteve associado ao aumento do peso fetal ao nascer e à incidência de macrossomia.

## INTRODUCTION

Weight gain during pregnancy has always been a matter of great concern for most women and obstetricians. This concern exists because gestational weight gain is related to many complications, both maternal and fetal.^1^^,^^2^ Macrosomia is a major fetal complication, consisting of cases of infants born weighing more than 4,000 g, regardless of the gestational age.^3^ This large weight is associated with complications for both the mother and the child. The most common complications in macrosomic fetuses include: increased risk of intrauterine death, hypertrophic cardiomyopathy, need for intensive care, shoulder dystocia, humeral and clavicle fractures, meconium aspiration, hypoglycemia, neonatal hyperbilirubinemia, paralysis of the facial and brachial plexus and obesity in childhood and adulthood.^4^^,^^5^^,^^6^^,^^7^ For mothers, the most common complications include: increased risk of cesarean section, cephalopelvic disproportion, prolonged labor, soft-tissue lacerations and postpartum hemorrhage.^4^^,^^6^

The incidence of fetal macrosomia ranges from 4.8% to 6.7% in Brazilian studies and from 12.8 to 37.4% in studies worldwide.^8^^,^^9^^,^^10^^,^^11^ This high incidence may be associated with several factors, such as advanced maternal age, multiparity, pregestational overweight and obesity, short stature, excessive gestational weight gain and gestational diabetes.^12^ Despite the numerous studies on the relationship between maternal and fetal weight gain conducted in developed countries, data in developing countries is scarce.^13^

## OBJECTIVE

The objective of this study was to investigate the association between gestational weight gain and fetal birth weight and the incidence of macrosomia, among pregnant women with an initially normal body mass index (BMI).

## MATERIALS AND METHODS

Data were gathered between January 2006 and December 2008. The study design was of cohort type, matched according to age. The study included 200 pregnant women who had been hospitalized for childbirth in two public maternity hospitals in Goiânia, Brazil: one of them secondary and the other, tertiary. The women were divided into two groups, of 100 individuals each. The group with excessive weight gain was composed of women who had gained 16 kg or more, while the group of normal weight gain consisted of women whose weight gain had been ³ 11.5 kg and < 16 kg during the same period.

To select the subjects, the researcher visited the two maternity hospitals every second day and analyzed the records of the hospitalized women. Those who met the inclusion criteria were interviewed before they completed 48 hours of puerperium. Information regarding the women’s pregnancies, such as gestational weight before the 14^th^ gestational week, final weight (after the 37^th^ week), height, age and fetal birth weight were obtained from all delivery documents. Information regarding the puerperal women’s dietary behavior, socioeconomic characteristics (education and income) and lifestyle were obtained from a face-to-face interview.

The sample size was calculated taking a confidence interval of 95% and test power of 80%. The expected frequency of individuals exposed to an excessively caloric diet in the control group (normal weight gain) was taken to be about 20%, while in the case group (excessive weight gain), the prevalence rate was taken to be four times greater. Therefore, it was stipulated that it would be necessary to interview 90 patients, divided into two groups of 45 each. Another 10% was added to account for possible losses, thus making up a total of approximately 100 patients required in order to identify this difference. However, because multiple factors have been correlated with weight gain, we decided to include twice the number of individuals, i.e. 200 women, in order to maximize the capacity of the multivariate analysis.

The puerperal women included in this study had been classified as eutrophic at the beginning of their pregnancies, in accordance with their pre-gestational BMI, which ranged between 18.5 kg/m^2^ and 24.9 kg/m^2^. They were aged between 20 and 40 years, and had their delivery between the 37^th^ and 42^nd^ gestational week. The gestational age was estimated based on the last menstruation period and the first trimester ultrasound examination. Women were excluded from the study if they were of indigenous ethnicity (due to different dietary behavior and lifestyles, and also because they are a minority in the city of Goiânia) and if, at any time during pregnancy, they presented any chronic hypertensive disease, diabetes, lupus, heart disease, preeclampsia or twin pregnancy.

All the puerperal women were informed about the objectives of the study and the procedures that would be performed. The subjects participated voluntarily and signed an informed consent form after being informed about the nature and objectives of the study. The study had previously been approved by the Research Ethics Committee of the Teaching Hospital of the Federal University of Goiás (Universidade Federal de Goiás, UFG), under number 063/2005.

The researcher interviewed the participants using a structured questionnaire, which contained questions regarding the women’s social, economic, demographic and lifestyle characteristics, such as age (in completed years on the date of the interview), education (in completed years on the date of the interview), income (expressed in Brazilian reais and as minimum salaries), number of pregnancies, parity (number of children that had been born), abortions/miscarriages, birth interval (time between the date on which the last child was born, taking into account miscarriages, and the date of the last period), date of the last period (in months), drug use, smoking during pregnancy and fetal birth weight (the weight noted on the child’s or mother’s medical record). Fetal birth weight was classified as follows: low birth weight (< 2,500 g); normal weight (³ 2,500 g and < 4,000 g); and macrosomia (³ 4,000 g).

Three of the subjects reported not knowing their family income. The minimum salary was R$ 350.00 until April 2007, R$ 380.00 until March 2008, and R$ 415.00 until the end of the year when the data gathering was completed.

In the anthropometrical evaluation, the following factors were analyzed: pregestational weight, i.e. the weight measured and registered on the mother’s card before the 14^th^ week of pregnancy, expressed in kilograms; height expressed in centimeters; final weight (measured after the 37^th^ week of pregnancy); pregestational BMI, calculated based on the relationship between the woman’s pre-gestational weight and height squared, expressed in kg/m^2^, and classified in relation to the normal range proposed by the World Health Organization (WHO), i.e. between 18.5 and 24.9 kg/m^2^; and final BMI (calculated based on the relationship between the woman’s final weight and height squared).^14^

The data gathered were typed and filed as spreadsheets using Microsoft Excel 2003, and were analyzed using the Statistical Package for the Social Sciences (SPSS) version 17.0. The Kolmogorov-Smirnov test was performed to evaluate whether the numerical variables presented normal distribution, and the distribution was considered to differ from the normal curve when P < 0.05. For variables with normal distribution, the mean, standard deviation and Student’s t test were used. For variables with distribution differing from normal, the median, interquartile range and Mann-Whitney test were used. The frequency and the chi-square test (c²) or Fisher’s exact test were used for categorical variables. The independent numerical variable (maternal weight gain) was correlated with fetal birth weight through Spearman’s rank correlation coefficient, since gestational weight gain presented a distribution differing from the normal curve.^15^

## RESULTS

Table 1 presents the subjects’ ages, education levels, economic characteristics (per capita income), reproductive behavior (number of pregnancies, parity, abortions and birth interval) and lifestyles (duration of smoking habit and number of cigarettes per day). None of the patients reported using drugs during pregnancy. Table 2 presents the frequency of miscarriages and smoking during pregnancy in the study population. The study groups were considered to be similar in relation to these factors.

**Table 1.** Social, economic, demographic and lifestyle characteristics of the postpartum women, compared between the cohorts. Goiânia, 2006-2008

Variables	Excessive weight gain		Normal weight gain		P^†^
	Median	IQR^*^	Median	IQR^*^	
Age (years)	25.00	(22.00 - 28.75)	24.00	(22.00 - 27.75)	0.52
Education (years)	10.50	(9.00 - 12.00)	11.00	(9.00 - 12.00)	0.77
Income per person (MS^‡^)	0.85	(0.56 - 1.20)	0.80	(0.48 - 1.18)	0.37
Duration of smoking (months)	0.00	(0.00 - 0.00)	0.00	(0.00 - 0.00)	0.24
Number of cigarettes/day	0.00	(0.00 - 0.00)	0.00	(0.00 - 0.00)	0.22
Number of pregnancies	2.00	(1.00 - 3.00)	2.00	(1.00 - 3.00)	0.61
Parity	2.00	(1.00 - 3.00)	2.00	(1.00 - 2.00)	0.45
Number of abortions	0.00	(0.00 - 0.00)	0.00	(0.00 - 0.00)	0.90
Birth interval (months)	27.00	(0.00 - 60.75)	22.50	(0.00 - 47.75)	0.34

^*^IQR = interquartile range; ^†^Mann-Whitney test; ^‡^minimum salaries.

**Table 2.** Frequency of miscarriages and smoking during pregnancy in the study population

	Excessive weight gain (%)	Normal weight gain (%)	χ**²**	P*
Smoking				
Yes	13 (13%)	8 (8%)	1.33	0.25
No	87 (87%)	92 (92%)		
Number of miscarriages				
0	79 (79%)	80 (80%)	0.24	0.89
1	17 (17%)	15 (15%)		
³ 2	4 (4%)	5 (5%)		

*Chi-square test (χ²).

The number of women classified as physically active was 28 (28%) in the group with excessive gestational weight gain and 31 (31%) in the group with normal weight gain (P = 0.64).

The anthropometric data are presented in Table 3. The variables of total weight gain and final BMI presented significant differences (P < 0.01), as expected, because the postpartum women were divided according to their weight gain for their participation in the study (excessive weight gain, ³ 16 kg, and normal weight gain, from ³ 11.50 kg to < 16 kg).

**Table 3.** Anthropometric profile of the cohorts of postpartum women. Goiânia, 2006-2008

Variables	Excessive weight gain		Normal weight gain		P^†^
	Mean	(?) SD^*^	Mean	(?) SD^*^	
Initial BMI^‡^ (kg/m²)	21.68	1.83	21.34	1.67	0.16
Final BMI^‡^ (kg/m²)	29.22	2.38	26.61	1.76	< 0.01
Total weight gain (kg)	19.36	3.39	12.91	1.40	< 0.01

*SD = standard deviation; ^†^Student’s t test; ^‡^BMI = body mass index (weight/height²).

**Figure 1.**
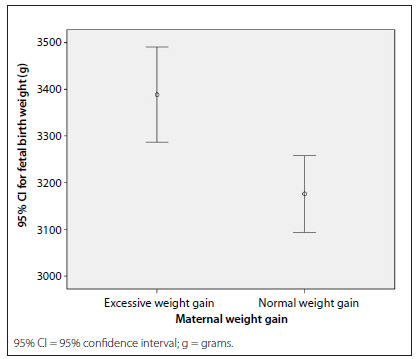
Comparison between mean fetal birth weights according to the categories of maternal weight gain during pregnancy, i.e. excessive weight gain: 3,388.83 g (? 514.44 g) and normal weight gain: 3,175.86 g (? 413.70 g) (P < 0.01).

Fetal birth weight was associated with maternal weight gain, as observed in Figure 1**.** The mean fetal weight at birth was 3,388.83 g (± 514.44 g) in the group with excessive gestational weight gain and 3,175.86 g (± 413.70 g) in the group with normal weight gain (P < 0.01).

**Figure 2.**
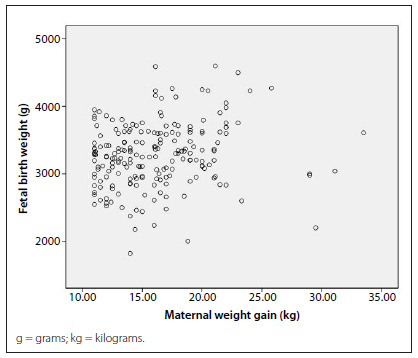
Correlation between fetal birth weight and the total weight gain during pregnancy, in the cohorts of puerperal women studied.

Fetal weight at birth was also correlated with total weight gain during pregnancy through Spearman’s rank correlation coefficient (0.19; P < 0.01), which is presented in Figure 2**.**

There were 13 cases (6.5%) of macrosomia in the study population, and all cases of macrosomia occurred in the cohort with excessive gestational weight gain. The association between macrosomia and total maternal weight gain through Fisher’s test is presented in Table 4. The relative risk (RR) could not be calculated because the incidence of macrosomia in the group with normal weight gain was zero. On the other hand, the incidence of low fetal weight was similar in the two study cohorts; i.e. four cases (4%) of excessive maternal weight gain, and five cases (5%) of normal weight gain (P = 0.73).

**Table 4.** Incidence of macrosomia in the cohorts

Variables	Macrosomia	Normal birth weight	P*
Excessive weight gain	13 (13%)	87 (87%)	< 0.01
Normal weight gain	0 (0%)	100 (100%)	

*Fisher’s exact test.

## DISCUSSION

The general incidence of macrosomia was 6.5%, which is in agreement with the results from other previous studies in Brazil. A cohort of 230 pairs of mothers and children living in Rio de Janeiro was evaluated between 1999 and 2001, and the incidence of macrosomia was 4.8%. Another cohort with 195 mothers attended at a primary healthcare unit between 2005 and 2007, also in Rio de Janeiro, presented a 6.7% incidence of fetal macrosomia.^9^^,^^10^ There has been an increase in the incidence of fetal macrosomia over recent decades.

Studies in other countries have reported high incidences of fetal macrosomia. A study published in 2004 on a cohort of 9788 women in California showed an 11% incidence of fetal macrosomia. Another cohort in Denmark with 43,561 women indicated that fetal macrosomia increased from 16.7% in 1990 to 20% in 1999. A cohort in Tunisia with 350 puerperal women found that fetal macrosomia increased by 15.8% between 2002 and 2003.^16^^,^^17^^,^^18^

The increasing incidence of fetal macrosomia in Brazil can be explained by the nutritional transition that has been taking place with the development of this country.^19^ Taking this context into consideration, it can be expected that the incidence of fetal macrosomia in Brazil may further increase, just as in developed countries. Therefore, fetal macrosomia has become a factor of great importance and must be monitored in order to minimize its deleterious consequences.

In this study, we observed that there was a significant correlation between maternal weight gain and fetal birth weight. This association was also observed in another study, with a cohort in Rio de Janeiro, from 1999 to 2001, which evaluated the relationship between various factors such as multiparity, pregestational overweight or obesity, advanced maternal age, prolonged gestational age and excessive gestational weight gain with the manifestation of fetal macrosomia. In that study, only excessive gestational weight gain was significantly associated with macrosomia.^9^

Other studies have found a relationship between fetal macrosomia and other factors associated with pregnancy, such as parity.^19^^,^^20^ Women with many children were found to have a greater chance of having macrosomic babies than nulliparous women did. In our study, the number of pregnancies and parity in the cohorts were similar.

The issue of dietary behavior during pregnancy remains a very complex subject, with no consensus up to now. A study conducted by Kramer et al. in 1998 showed that, during pregnancy, the quality of the food consumed is more important than the quantity.^20^ Another study by Moses et al. in 2006 found that women who had diets with high glycemic levels had a higher incidence of babies with macrosomia.^21^ The study by Denguezli et al. in 2009 showed that carbohydrate consumption higher than the recommendations was related to higher fetal birth weight.^15^ Therefore, dietary behavior during pregnancy is a subject that calls for further studies in order to reach a consensus and hence enable improvement of the level of guidance provided to pregnant women, and consequently avoid the undesired effects of excessive weight gain. We believe that it is possible to undo the undesirable association between weight gain and macrosomia through quality prenatal care, in which women are given guidance regarding healthy dietary behavior with appropriate quantities.

## CONCLUSIONS

In the present study, it was found that there was a direct relationship between maternal weight gain and fetal birth weight, i.e. mothers who gained more weight during their pregnancy had heavier babies, with higher incidence of macrosomia.
